# Recent Trends in the Development of Ecological Models Applied on Aquatic Ecosystems

**DOI:** 10.1100/tsw.2002.89

**Published:** 2002-03-12

**Authors:** S.E. Jorgensen

**Affiliations:** DFH, Institute A, Environmental Chemistry, University Park 2, Copenhagen East, 2100, Denmark

**Keywords:** aquatic ecosystems, machine-learning methods, artificial neural networks, genetic algorithms, object-oriented programming, structurally dynamic modeling

## Abstract

This paper presents an overview of the application of models on aquatic ecosystems. More than 17% of the models published in the focal journal in the field, Ecological Modelling, are aquatic ecosystem models. An increasing number of papers are dealing with the theoretical aspects of modeling – new modeling approaches and techniques, how models can be used to reveal ecosystem properties, and how models can better reflect the properties of ecosystems. This development implies that today we have more types of models. The characteristics, the advantages, and the disadvantages of these model types are presented briefly. The selection criteria for the presented model types are discussed, and the application of these types to models for aquatic ecosystems is reviewed. A recent improvement in model calibration of particular interest for aquatic ecosystems is presented, and the perspectives resulting from this new calibration procedure and from possible hybrids of the presented model types are discussed.

## INTRODUCTION

The journal *Ecological Modelling* had its 25-year anniversary in 2000, which was celebrated at a so-called ecosummit meeting with the participation of several Elsevier journals in the field of ecology. The first issues of this journal were published in 1975. It was very difficult to get enough publications for the first volume, although originally only 320 pages were published per year. Today, *Ecological Modelling* publishes around 3600 pages annually, and we have enough material for about twice as many papers , because ecological modeling is a well-recognized and widely-used tool today in environmental management and ecosystem research. To date, 55 countries have published in the journal, although most of the papers have come from western Europe (EU plus Switzerland and Norway) (47%), the U.S. (33%), and Japan (9%). But recently several papers from China have been submitted to the journal. It clearly can be concluded that models are used increasingly as tools both in management and in understanding the properties and reactions of ecosystems on a system level.

In the following section, we look at the trends in the development of ecological models by use of the publishing statistics of the journal *Ecological Modelling*. It is clear from the statistics that we have a wider spectrum of model types today. These model types are presented in the third section. The recently developed structurally dynamic model type has particular interest for aquatic ecosystems because they have usually a more rapid change in structure, even from season to season — the so-called succession. This problem implies that a calibration with fixed parameters independent of the time is sometimes extremely difficult (as discussed in the sixth section). This model type has mainly been applied on aquatic ecosystems. Obviously, a wider spectrum of model types implies that the selection of the best type for a given case study becomes more difficult. Therefore, the fifth section is devoted to discussing the selection of model type and which model type we mainly apply for aquatic ecosystems. The sixth section presents the latest improvements in the calibration procedure, based on the same theoretical approach as the structurally dynamic model. The improvements also solve the calibration problem associated with the succession, mentioned above. The last section summarizes the trends and perspectives in the present development of ecological modeling.

## TRENDS BASED ON THE PUBLISHING STATISTICS OF *ECOLOGICAL MODELLING*

The statistics on the papers published in the journal in the last 25 years can be used to indicate the trends in the development of the field of ecological modeling (see also Table 1):

1. The number of papers focusing on terrestrial problems is still increasing. In the last 3 years, 23% of the published papers were concerned with terrestrial problems.
2. The number of papers dealing with aquatic problems seems to have stabilized around 17%, which is also the average for the 25 years, while it was 30% in the first 5 years. However, the number of papers published in *Ecological Modelling* is 12 times greater today than 25 years ago.
3. In the last 3 years, 24% of the papers have treated theoretical aspects: modeling methods and modeling techniques (17%), use of models to reveal ecosystem theory (models used in an ecological system context) (4%), and use of thermodynamics to understand ecosystems (3%). Use of models to assess ecosystem health or integrity is a new topic in this group of modeling papers. We will probably see many more papers dealing with this application of models in the future, as it is a new line of environmental management that often will be or must be based on models.

The many papers on new modeling approaches indicate that we can expect more clear and rigorous modeling procedures and techniques around the corner. We have needed a general "model" of modeling for many years, but it does not exist yet. Each modeler uses his own model, but a *general* agreed procedure and technique should be developed.
4. Several countries that have never published before in *Ecological Modelling* have submitted papers during the last 3 years: Estonia, Chile, Costa Rica, Singapore, Oman, Puerto Rico, Philippines, Iceland, Turkey, and Yugoslavia. Ecological modeling is obviously becoming a global activity. A total of 55 countries have published in *Ecological Modelling*. However, more than 100 nations still have not published in the journal. Models are applied increasingly in environmental management, and environmental issues are of global interest. The basis for a more global coverage should therefore be present. The author of this paper has given short courses (duration 3 days to 2 weeks) in modeling in 28 countries. Recently, an Internet course of ecological modeling (www.enviromod.subnet.dk, or Elsevier's homepage for *Ecological Modelling*) has been launched, and it has already found a wide interest.

5. The trend in ecological modeling is toward application of a broader spectrum of modeling approaches: for instance, artificial neural networks, fuzzy modeling, and what is called structurally dynamic modeling, which is able by a goal function to describe the current changes in the properties of the dominant species. We see also more integrated models: models that integrate land use with pollution in aquatic ecosystems, landscape models, and economic ecological models. Furthermore, the journal has the intention to facilitate the access of the readers to the models published in the journal. Shortly, it will be possible to download the published models from the Internet.

**TABLE 1**
**Development in Model Topics**

| | % Allocation on Topics | | | |
|---|---|---|---|---|
| **Model Topics** | **1975–1979** | **1992–1996** | **1997–1999** | **Total** |
| Aquatic ecosystems | 30 | 13 | 17 | 17 |
| Global and climate | 3.2 | 4.1 | 3.4 | 3.7 |
| Population dynamics | 12 | 17 | 16 | 16 |
| Theoretical papers | 17 | 27 | 24 | 25 |
| ***Management*** | ***10*** | ***5*** | ***3.1*** | ***4.9*** |
| Ecological economics | 2.1 | 0.8 | 1.4 | 1.0 |
| Ecotoxicology | 0.1 | 6.2 | 5.5 | 5.5 |
| Terrestrial ecosystems | 6.0 | 19 | 23 | 18 |
| Waste water + air pollution | 3.1 | 0.9 | 0.3 | 1.3 |
| Other topics | 12 | 7 | 6 | 7 |

All in all, it can be concluded that models of aquatic ecosystems are still dominant and that new model types offer new possibilities for utilization.

## A SHORT PRESENTATION OF RECENTLY DEVELOPED MODEL TYPES

### Machine Learning

The applications of machine learning in the development of ecological models are in their infancy. There are probably a number of possible applications in ecological modeling that would improve our models, particularly in their ability to make more accurate predictions. Only imagination sets the limits for the use of machine learning in ecological modeling. Let us mention a few possible applications to illustrate this model type:

- A knowledge base is used to select more certainly and faster than today the most appropriate model structure from knowledge about the available data.
- Knowledge of relations between forcing functions and some key state variables on the one side, and the most crucial parameters on the other, is used to vary the parameters according to the variations of forcing functions and key state variables. By this method we can develop a structurally dynamic model.
- Basic physical, chemical, and *ecological principles* are used to increase the robustness, explanation capability, and verifiability of the model.
- Artificial neural networks also have been applied in ecological modeling.

Usually, a three-layered neural network is applied, with one input layer, one hidden layer, and one output layer. The input layer contains the factors that are of importance for the modeling result included in the output layer. The hidden layer encompasses the equations that can be used to relate the inputs to the outputs. The equations may be based on statistics, causal relationships, or any type of knowledge about the focal system, or a combination of the three. A set of observations is used to "learn" the right parameters or test alternative equations, etc., while an independent set is used to test the validity of the model — in principle this is not different from other modeling approaches. The difference is that the model structure facilitates current improvement when new observations are available to improve the relationships in the hidden layer.

Much of the data collected by ecologists exhibit a variety of problems, including complex data interactions and nonindependence of observations. Machine-learning methods have shown a good ability to interpret complex ecological data sets and synthesize the interpretation in the form of a model. The resulting synthesis, the model, cannot replace our dynamic modeling approach, which has a high degree of causality and therefore generates *general knowledge and understanding,* but the machine-learning methods may be considered as supplementary modeling methods that often are able to utilize the data better than dynamic models.

Two machine-learning methods will be presented in more detail here: artificial neural networks and the application of genetic algorithms.

Artificial neural networks (ANNs) are excellent tools to analyze a complex data set and are in most cases superior to statistical methods that attempt to do the same job. The genetic algorithms can be used to generate rules that will increase our understanding of ecosystem behavior and therefore facilitate modeling in general. These methods have a very high potential to be used in connection with dynamic models to improve submodels based on too weak knowledge or to introduce additional constraints on dynamic models (for instance, the use of a goal function; see below under Structurally Dynamic Modeling).

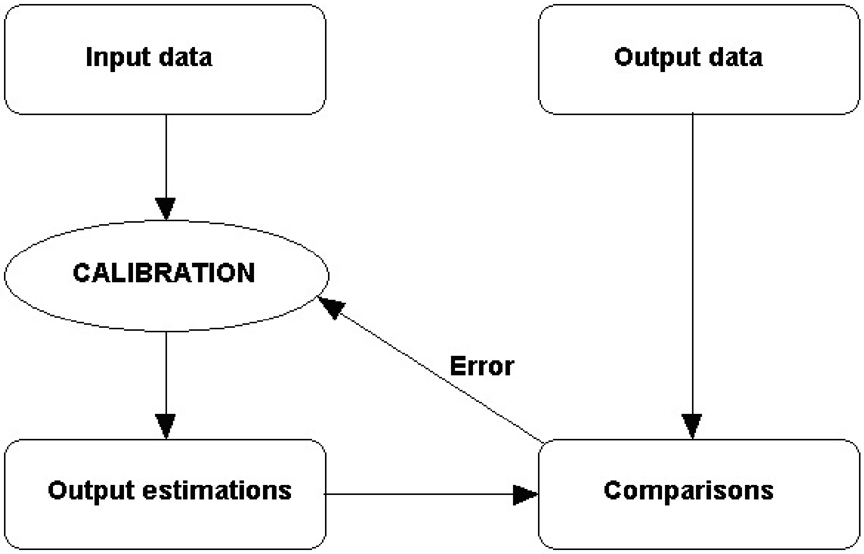

**FIGURE 1.** The diagram shows how data are used to establish the model calibration. The goal of the learning is to find a model that will as correctly as possible associate the input with the output data.

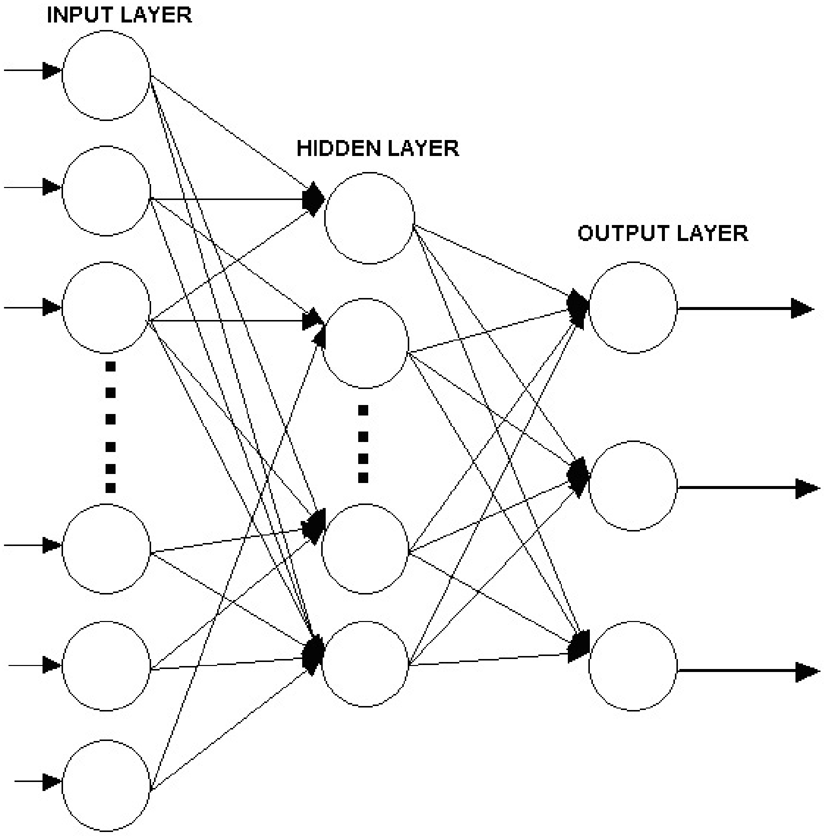

**FIGURE 2.** Illustration of a three-layered neural network with one input layer, one hidden layer, and one output layer.

## *Artificial Neural Networks*

Artificial neural networks (ANNs) are developed as models of biological neurons. They have found a wide application in science because of their power to interpret data. During the last decade, they have been used increasingly in ecological modeling; see, for instance, the review by Lek and Guégan[1].

The two most applied ANNs in ecological modeling are back propagation neural networks (BPNs) and self-organizing mapping (SOM).

The BPN architecture is a layered-feed neural network. The information flows from the input layer to the output layer through the hidden layer (see Fig. 2). Nodes from one layer are connected to all the nodes in the next layer, but there are no connections between nodes within one layer.

Fig. 3 shows the basic processing element, a neuron, with its connections. Each neuron is numbered. The inputs are indicated as $x_1$, $x_2$, $x_3$,…$x_n$ and are associated with a quantity called weight or connection strength, $w_{j,1}$, $w_{j,2}$, $w_{j,3}$,…$w_{n,j}$, for the input to the jth neuron. Both positive and negative weights may be applied. The net input, denoted activation, for each neuron is the sum of all its inputs multiplied by their weights + z, a bias term that may be considered the weights from supplementary input units:

$$a_j = \sum w_{ji} x_i + z \qquad (1)$$

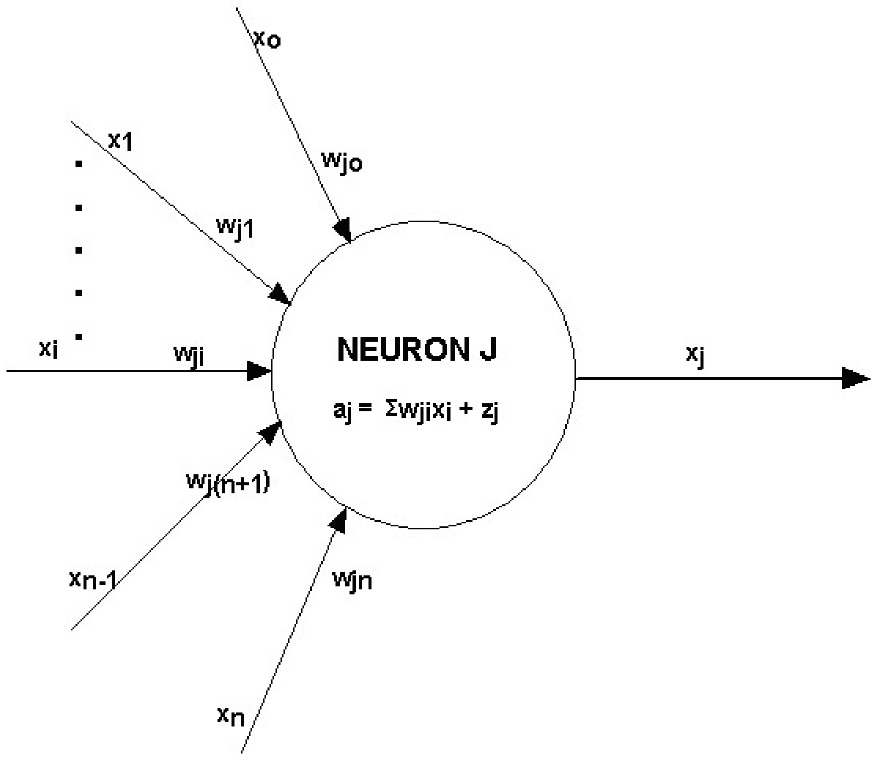

**FIGURE 3.** The basic processing element (a neuron) in a network receives several input connection values associated with a weight. The resulting output value is computed in accordance with the presented equations (see the text).

The output value, $x_j$, called the response, can be calculated from the activation of the neuron:

$$x_j = f(a_j) \tag{2}$$

Many functions may be used: e.g., a linear function, a threshold function, and most often a sigmoid function.

The weights establish a link between the input data and the associated output. They contain therefore the knowledge of the neural network about the problem–solution relationship. The forward-propagating step begins with the presentation of the input data to the input layer and continues as activation-level calculations propagate forward to the output layer through the hidden layer using the equations presented above. The backward propagation step begins with the comparison of the network output pattern to the observations (the target values). The error values (the differences between outputs and target values), d, are determined and are used to change the weights, starting with the output layer and moving backward through the hidden layer.

## *Genetic Algorithms*

Genetic algorithms provide an alternative approach to model (submodel) selection. They develop iteratively a set of rules that help to explain the relationships between variables or attributes included in the data set. Several genetic algorithms are available, but they all have more or less the same features.

A typical illustration of the use of genetic algorithms in ecological modeling can be found in Recknagel et al.[2] They are, for instance, able to set up predictive rules (threshold values for concentrations of nitrogen and phosphorus and temperature) for the presence and approximate concentration of *Microcystis* based upon data from Kasumigaura Lake. These rules are applied in a eutrophication model for Kasumigaura Lake to describe the succession of species or the change in species composition resulting from change in the variables included in the rules.

The application of genetic algorithms in ecological modeling seems promising. They probably could be used much wider to select submodels and to develop a more streamlined application of goal functions in structural dynamic models. A combination of rules generated by genetic algorithms and the use of goal functions for the development of better structural dynamic models will probably be seen in the near future.

## Object-Oriented Programming

The central concept of object-oriented programming (OOP) is the concept of class, which describes both the structure of an object and a set of procedures for initializing and using it in the model. One obvious example of a class is the definition of a population, which is the basic building block for many ecological models. Populations are characterized by variables such as mean size, age, and number, and exhibit processes such as reproduction, growth, mortality, etc. Each type of population is unique, although there are many similarities, such as the above-mentioned processes. We can therefore treat different classes of populations accordingly and need only to add those particular features that need to be different in the model context.

The OOP defines different processes in different modules that can be used in the various classes. It is possible to have several different versions of the processes. The program can, for example, have different growth routines. The growth routine is inherited (see below for further explanation) from the class but can also be redefined to cover all other growth expressions. This means that we can use the fact that every population is represented by a class that includes a growth procedure without knowing the precise details of how growth is calculated, and it means that changes in the growth procedure for certain classes do not require changes in the overall structure of the ecological model. This leads naturally to the concept of hierarchy. It is often difficult in ecological modeling to draw the line between processes relevant to the model and those that operate on a different level and should not be included. OOP offers a mechanism that lets us hide this more detailed information on the internal description of objects so that we can use it without having to describe it explicitly in our model.

The hierarchy can be constructed, for instance, by describing first populations, then plants, then classes of plants (such as algae), and finally species. This gives a hierarchy of four classes, each based upon the one above it. At each stage, we can add and modify information appropriate to the level of description by application of what is called inheritance. Plants may include, for example, two parameters beyond those shared by all populations, such as growth rate and carrying capacity. Algae then share these properties but also have nutrient limitation characterized by a half saturation constant, so that growth has to be redefined in the algae class. The classes for species finally may give information on the settling rate, which in this case will be different for the various species, while all species of algae share the common properties of algae, of plants, and of populations. This system gives the advantage that changing an inherited method automatically changes all of the classes that inherit that method. Fig. 4 illustrates class hierarchy for an object-oriented model of a cotton plant and associated insect pests.

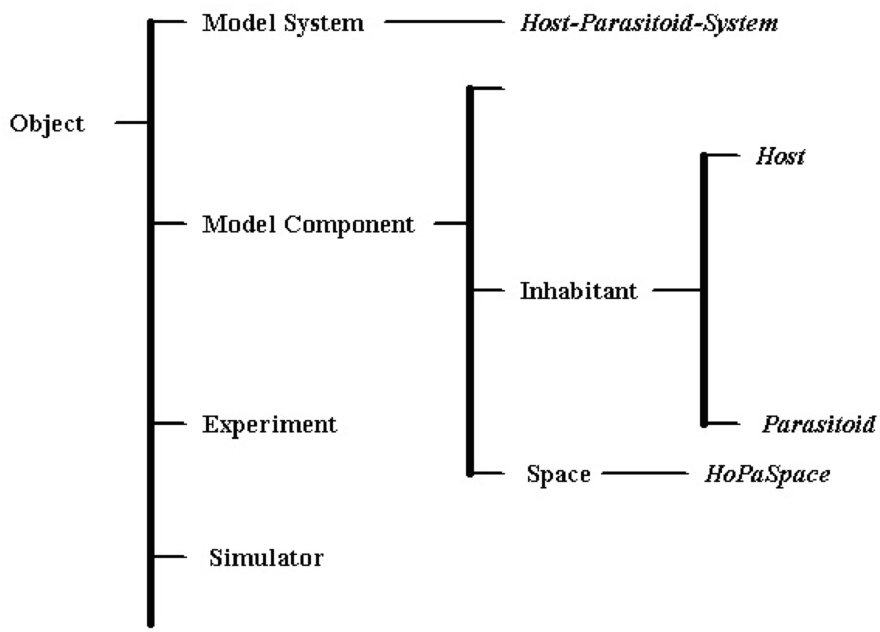

**FIGURE 4.** Class hierarchy for an object-oriented simulation of a cotton plant and associated insect pests. Reproduced from Baveco and Lingeman[3] with permission. HoPaSpace covers the spatial distribution of properties.

OOP has only recently received extensive notice even though it has evolved over several decades (see Muetzelfeldt[4] and Meyer and Pampagnin[5]). Today there are many languages, for instance, C++, that offer support for OOP. It is expected that it will be increasingly used during the coming years as a more convenient method of programming ecological models.

OOP offers many advantages for developers of ecological models. First of all there is a close connection between objects and natural groupings. The concept of inheritance is directly borrowed from biology. OOP makes it possible to develop models that are simpler to interpret for the modeler and that can be modified easily and refined very efficiently. Examples of object-oriented models in ecology can be found in the following references: Sequeira et al.[6], Baveco and Lingeman[3], and Silvert[7].

## Individual-Oriented or Individual-Based Models

Individual-oriented or individual-based models (IBMs) attempt to account for the enormous variability among individuals usually represented in our models by one state variable. Individual-oriented modeling acknowledges two basic ecological principles that are violated in most ecological models, namely the individuality of individuals and the locality of their interactions. Without an inequality among population members, contest competition is not possible; and individuals process local information!

The advantages of this modeling approach are obvious. Still, the defense for the approach is often made as a confrontation of holism vs. reductionism, which is a misunderstanding. Ecosystems *have* the properties of the individuality of individuals and the locality of their interactions. There is also no doubt that these properties are significant in a number of relations, and they should therefore be accounted for in our models. This still does not change the fact that the ecosystem as a system has some properties that cannot be deducted from the sum of the components, and that the model (IBM or not) still cannot account for more than a tiny fraction of the details of the real ecosystems. We are therefore always forced to consider which simplification can be made and which cannot be made in each concrete modeling situation. There are indeed situations where we cannot exclude the individuality and the locality but where we need these properties as a core of our model. An average state variable cannot be used in most cases to represent a population, as the core relationships are not linear.

The individuality of individuals can in principle be considered by three methods: (1) Leslie matrix models, (2) i-space configuration models, and (3) relating the properties of individuals to one or at the most a few core state variables such as body size, length, weight, or age. I-space configuration models use continuous distribution functions. The change at one point along the size continuum is described by a mathematical equation (see the example in DeAngelis and Gross[8]). Benjamin[9] gives a typical example, where the crop growth is determined by the spatial planting pattern and the competition for light, which is considered the limiting factor for growth. The application of the third method, i.e., finding a core variable to which other variables can be related, is completely according to the presentation of relations between parameters and body size. Wyszomirski et al.[10] use the size distribution in crowded and uncrowded monocultures to determine and explain the growth pattern.

A very good overview of individual-based models in ecology is given in DeAngelis and Gross[8], where many illustrative examples can be found. *Ecological Modelling* had a special issue in 1999 on “Individual-Based Modeling in Ecology”.

## Fuzzy Knowledge–Based Models

Ecological data bear a large inherent uncertainty due to inaccuracy and lack of sufficient knowledge about parameters and state variables. On the other hand, semiquantitative model outputs might be sufficient in many management situations. Fuzzy knowledge–based models can be applied in such situations. Zadeh[11] proposed a method to process imprecise knowledge by using a changed membership function. The membership function takes only two values: one when it belongs to the set and zero when it does not. The shape of the fuzzy set membership can be linear or trapezoidal, as shown in Fig. 5.

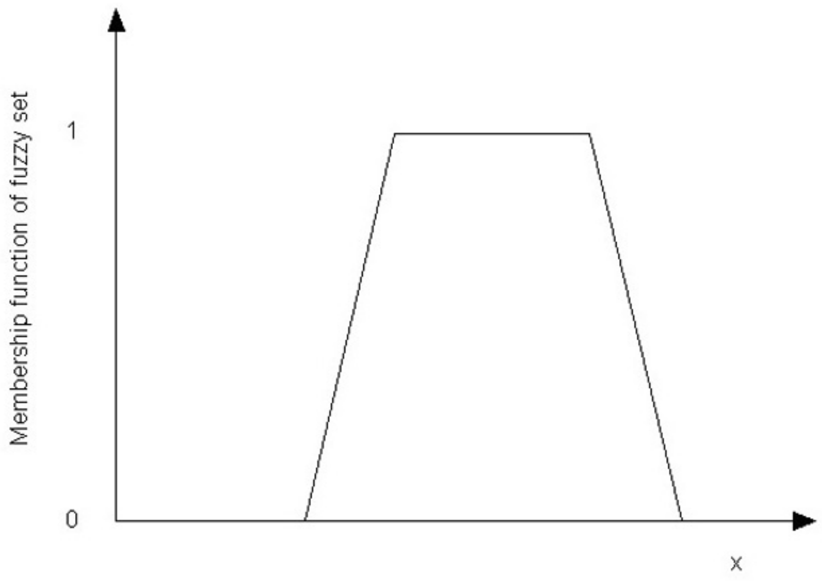

**FIGURE 5.** A trapezoidal fuzzy set F in x.

Ecologists often use natural language for describing their knowledge about ecosystems, such as, "if vegetation is low, and the population of larks is very high, and vegetation density is smaller than standard, then the number of territories for the larks will be high." These linguistic rules can be defined in the form of fuzzy sets (see Zimmermann[12]). If A and B are fuzzy sets where we know that if A is true B is also true, the problem is: how do we account for A' that fulfills the premise only partially? To calculate the conclusion B' we have to set a relationship based upon approximated reasoning rules as follows:

$$B' = A' \text{€} R \qquad (3)$$

where € is a composition operator and R is a fuzzy relation. Fuzzy set theory formulates many different forms of what are called composition operators and methods for the calculation of fuzzy relations.

Development of a fuzzy knowledge–based model requires first the determination of the model structure, i.e., input and output variables, the number of submodels, connection between submodels, etc. Then the knowledge base is constructed by determination of linguistic rules. Fuzzy sets can then be defined to describe the linguistic rules. The major problem of fuzzy modeling is to find an appropriate set of rules describing the modeled system. They must be taken directly from an expert's experience. The set of rules should be complete and provide correct answers for every possible input value. Therefore the sum of all input values (union of fuzzy sets) should cover the value space of all input variables.

The set of linguistic rules, definition of fuzzy sets, and facts (data) compose the main parts of the fuzzy model and are called the fuzzy knowledge base (see Fig. 6). A fuzzy inference method is used to process this knowledge and compute output values corresponding to the input values. The input values can be numerical or fuzzy sets. Linguistic terms are also allowed as inputs. The output values have the form of fuzzy sets, which can be translated into numerical values (by a so-called defuzzification process) or approximated to one of the linguistic terms that we have defined for the output variables (see Fig. 6).

Only a few examples of fuzzy knowledge–based ecological models have been published, but this is probably a method that will have an increased use in the near future, because it is a very appropriate method for a number of ecological problems where our knowledge is only semiquantitative. Salski[13] has presented a very illustrative example that gives details about this modeling technique.

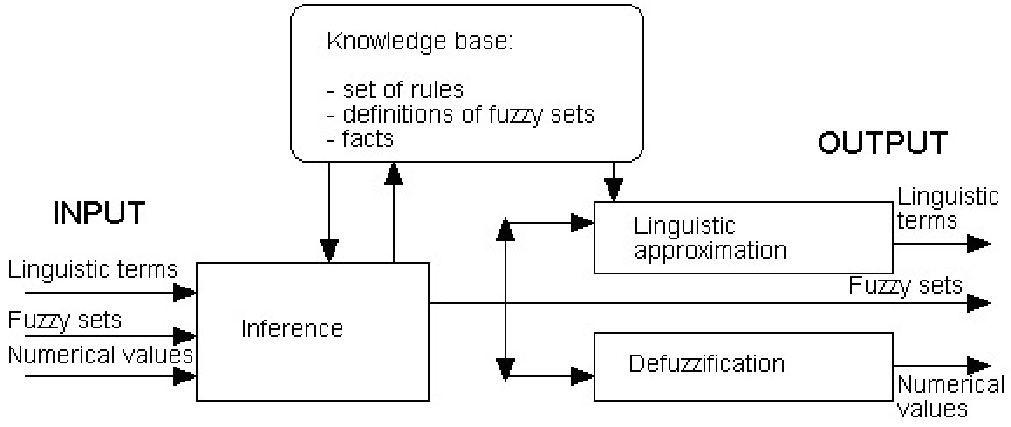

**FIGURE 6.** Information flow in the fuzzy knowledge–based model.

## STRUCTURALLY DYNAMIC MODELING (SDM)

If we follow the general modeling procedure, we will attain a model that describes the processes in the focal ecosystem, but the parameters will represent the properties of the state variables as they are in the ecosystem during the examination period. They are not necessarily valid for another period, because we know that an ecosystem can regulate, modify, and change them, if needed, as a response to the change in the prevailing conditions determined by the forcing functions and the interrelations between the state variables. Our present models have rigid structures and a fixed set of parameters, reflecting that no changes or replacements of the components are possible. We need, however, to introduce parameters (properties) that can change according to changing forcing functions and general conditions for the state variables (components) to continuously account for adaptation.

The needed type of model should account for the change in species composition as well as for the ability of the species, i.e., the biological components of our models, to change their properties, i.e., to adapt to the prevailing conditions imposed on the species. Models of this type are named structurally dynamic models (SDMs) to indicate that they are able to capture structural changes, as illustrated in Fig. 7. Evolution, on the other hand, is related to the genetic pool. It is the result of the relation between the dynamics of the external factors and the dynamics of the genetic pool. The external factors steadily change the conditions for survival, and the genetic pool steadily comes up with new solutions to the problem of survival.

The possible shifts in species composition are determined by the gene pool, which is steadily changed due to mutations and new sexual recombinations of genes. The development is, however, more complex, as the species can modify their own environment (see below) and thereby their own selection pressure. An arrow from “structure” to “gene pool” indicates the possibility that species, to a certain extent, can change their own gene pool.

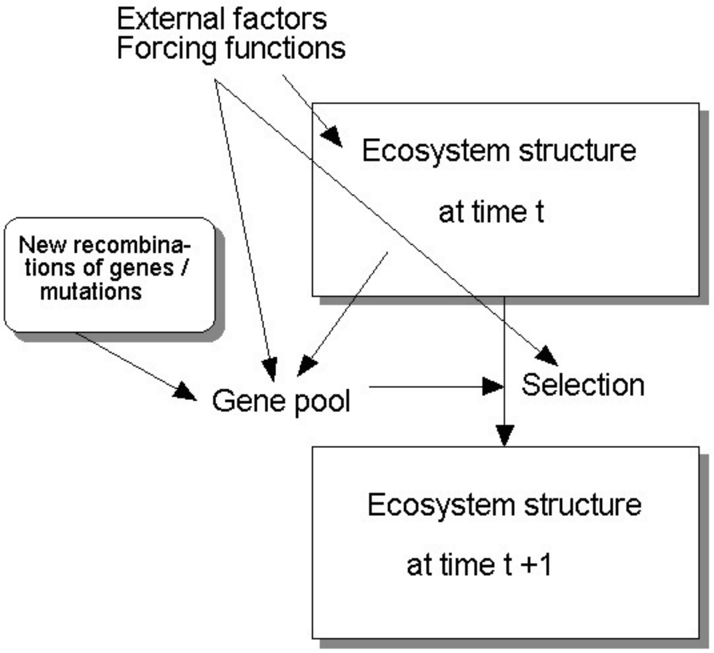

**FIGURE 7.** Conceptualization of how external factors steadily change the species composition.

SDMs also may be called the next or fifth generation of ecological models, to underline that they are radically different from previous modeling approaches and can do more, namely describe changes in species composition. SDMs are constructed by use of expert knowledge or goal functions, i.e., functions that describe continuously the development of models by optimization of the goals.

Several goal functions have been proposed, but only very few models have been developed that account for the change in species composition or for the ability of the species to change their properties within some limits.

Exergy has been used most widely as a goal function in ecological models, and a few of the available case studies will be presented and discussed below in this section. Exergy has two pronounced advantages as a goal function compared with entropy and maximum power: it is defined far from thermodynamic equilibrium, and it is related to the state variables, which are easily determined or measured. However, as exergy is not a generally used thermodynamic function, we need first to present this concept.

Exergy expresses energy with a built-in measure of quality like emergy. Exergy accounts for natural resources (see Jørgensen[14]) and can be considered as fuel for any system that converts energy and matter in a metabolic process (see Schrödinger[15]). Ecosystems consume energy, and an exergy flow through the system is necessary to keep the system functioning. Exergy measures the distance from the "inorganic soup" in energy terms, as will be further explained below.

Exergy, Ex, may be defined by the following equation:

$$Ex = To * NE = To * I = To * (Seq - S) \qquad (4)$$

where To is the temperature of the environment, I is the thermodynamic information, defined as NE, NE is the negentropy of the system, i.e., = (Seq – S) = the difference between the entropy for the system at thermodynamic equilibrium and the entropy at the present state.

It can be shown that exergy differences can be reduced to differences of other, better-known thermodynamic potentials, which may facilitate the computations of exergy in some relevant cases.

As can be seen, the exergy of the system measures the contrast, the difference in free energy between the system and the surrounding environment. If the system is in equilibrium with the surrounding environment, the exergy is zero.

Since the only way to move systems away from equilibrium is to perform work on them, and since the available work in a system is a measure of the ability, we have to distinguish between the system and its environment or thermodynamic equilibrium (alias the inorganic soup). Therefore it is reasonable to use the available work, i.e., the exergy, to express the distance from thermodynamic equilibrium. Let us turn to the translation of Darwin's theory into thermodynamics, applying exergy as the basic concept. Survival implies maintenance of the biomass, and growth means increase of biomass. It costs exergy to construct biomass, and biomass therefore possesses exergy, which is transferable to support other exergy (energy) processes. Survival and growth can therefore be measured by use of the thermodynamic concept of exergy.

Darwin's theory may therefore be reformulated in thermodynamic terms as follows: the prevailing conditions of an ecosystem steadily change, and the system will continuously select the species and thereby the processes that can contribute most to the maintenance or even growth of the exergy of the system.

Ecosystems are open systems and receive an inflow of solar energy. Solar energy carries low entropy, while the radiation from the ecosystem carries high entropy.

Notice that the thermodynamic translation of Darwin's theory requires that populations have the properties of reproduction, inheritance, and variation. The selection of the species that contribute most to the exergy of the system under the prevailing conditions requires that there are enough individuals with different properties so that a selection can take place — which means that the reproduction and the variation must be high and that, once a change has taken place due to better fitness, the change can be conveyed to the next generation.

Notice also that the change in exergy is not necessarily ≥0; it depends on the changes of the resources of the ecosystem. The proposition claims, however, that the ecosystem attempts to reach the highest possible exergy level under the given circumstances and with the available genetic pool ready for this attempt (see Jørgensen and Mejer[16,17]).

It is not possible to measure exergy directly, but it is possible to compute it if the composition of the ecosystem is known. Jørgensen and Mejer[17] have shown by the use of thermodynamics that the following equation is valid for the components of an ecosystem:

$$Ex = RT \sum_{i=1}^{i=n} (Ci * \ln (Ci/Ceq,i) - (Ci - Ceq,i)) \qquad (5)$$

where R is the gas constant, T is the temperature of the environment (Kelvin), Ci represents the ith component expressed in a suitable unit (e.g., for phytoplankton in a lake, Ci could be milligrams of a focal nutrient in the phytoplankton per liter of lake water), Ceq,i is the concentration of the ith component at thermodynamic equilibrium (which can be found in Morowitz[18]), and n is the number of components. Ceq,i is, of course, a very small concentration of organic components, corresponding to the probability of forming a complex organic compound in an inorganic soup (at thermodynamic equilibrium). Morowitz[18] has calculated this probability and found that for proteins, carbohydrates, and fats the concentration is about 10–86 µg/l, which may be used as the concentration at thermodynamic equilibrium.

The idea of the new generation of models presented here is to find continuously a new set of parameters (limited for practical reasons to the most crucial, i.e., sensitive parameters) that is better fitted for the prevailing conditions of the ecosystem. "Fitted" is defined in the Darwinian sense by the ability of the species to survive and grow, which may be measured by the use of exergy (see Jørgensen[19,20,21,22,23,24] and Jørgensen and Mejer[16]). Fig. 8 shows the proposed modeling procedure, which has been applied in the cases presented below.

An ecologically useful exergy index can be computed based on concentrations of chemical components, ci, multiplied by weighting factors, ßi, reflecting the exergy contents of the various components due to their chemical energy and the information embodied in DNA:

$$Ex = \sum_{i=0}^{n} \beta i c i \qquad (6)$$

Values for βi based on detritus exergy equivalents are available for a number of different species and taxonomic groups. The unit of detritus exergy equivalents expressed in g/l can be converted to kJ/l by multiplication by 18.7, which corresponds approximately to the average energy content of 1 g of detritus (see Morowitz[18]). The index i = 0 for constituents covers inorganic components, but in most cases these will be neglected, as contributions from detritus and living biota are much higher due to extremely low concentrations of these components in the reference system. Our exergy index therefore accounts for the chemical energy in organic matter plus the information embodied in living organisms. It is measured from the extremely small probabilities of forming living components spontaneously from inorganic matter. The weighting factors, βi, may be considered as quality factors reflecting the extent to which different taxa contribute to overall exergy. The exergy index calculated by Eq. 6 has been used successfully as a goal function to develop SDMs according to the procedure in Fig. 8. For further details on exergy and how Eq. 6 can be shown to be a reasonable approximation, see Jørgensen[14] or Jørgensen et al.[25].

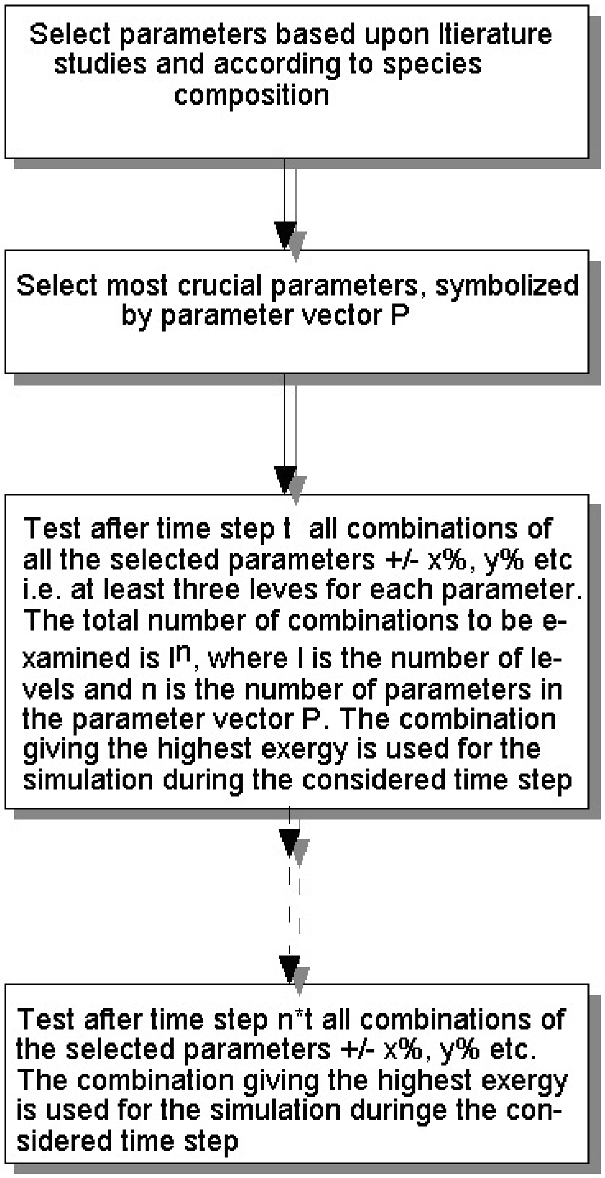

**FIGURE 8.** The procedure used for the development of SDMs.

## SELECTION OF MODEL TYPE

Today we have the possibilities to select among many new model types. The selection should reflect the problem, the ecosystem characteristics, and the available data.

If the model is developed on the basis of a database that has a limited quality and/or quantity, a model with relatively low complexity should be applied. A dynamic model is more demanding to calibrate and validate than a static model. Therefore, the latter type should be selected in a data-poor situation, provided of course that a description of the steady state is sufficient to solve the problem. Steady-state descriptions imply that an equation input = output for each state variable can be applied to find (estimate) one (otherwise unknown) parameter. A steady-state model can be developed and utilized to get a good overview of a pollution situation, even in a relatively data-poor situation.

Dynamic models are able to make predictions about the variations of state variables in time and/or space. However, many population-dynamic, biogeochemical, and ecotoxicological models apply differential equations, because the time variations are of importance.

Variation in both time and space require application of partial differential equations. The space variations may be considered by a discretization. The system can, for instance, be divided into boxes. Combinations of hydrodynamics and ecological models are typical examples of applications of partial differential equations.

Fuzzy models are employed when the observations used to develop the model are only indicated as ranges or classes (such as high, medium, and low), or by application of non-numeric natural language. The model results are interpreted in the same way, i.e., either as ranges or classes, but in many management and even research situations this is sufficient.

It is known that ecosystems are adaptable. The species can currently change their properties to meet changing conditions (i.e., change of forcing functions). If the changes are major, there may even be a shift to other species with properties better fitted to the emerging conditions. Models that account for the change of properties of the biological components have variable parameters and are described by nonstationary, time-varying differential equations. They are often called SDMs (for example, see Jørgensen[14,20]) because they are able to predict the changes in properties of the biological components. They are distributed models in the sense that the parameters are considered functions of time and space. But while distributed models in most cases are based on mathematical formulations of these functions when the model is developed, we will only use the term "structurally dynamic models" for models that can predict the changes of the structure (shifts of the properties, i.e., of the parameters). SDMs are an important recent development in ecological modeling, because the parameters found on the basis of the observations in the ecosystem under the present prevailing conditions cannot be valid when the conditions are changed due to the adaptation. Models without dynamic structure can therefore not give reliable prognoses if the forcing functions are changed significantly. The SDM approach has been applied to explain the failure and success of biomanipulation. This is also possible by catastrophe theory, but SDM can in addition explain the shifts in size of phytoplankton and zooplankton that take place simultaneously with the shifts from low to high phytoplankton concentration (see Jørgensen and de Bernardi[26]).

The parameter variation can also be determined by incorporation of knowledge (expert system, machine learning) of the relationships between forcing functions and the variation of relevant parameters. Reynolds[27] illustrates the application of this method. Relationships between wind exposure, depth, and nutrient concentrations on the one side and the dominant phytoplankton species on the other side are used to describe the change in species and thereby the parameter shifts. The variation can also be described by a goal function.

The variation of the focal parameters is determined by optimization of a defined function, for example, biomass or exergy. An illustrative example using exergy as a goal function was given above. When using this approach, it is often advantageous to apply the allometric principles. Most of the parameters that may change are expressed by size (length, volume). The goal function is then optimized by variation of the size as the only variable. The following procedure is applied: optimization of the goal function by varying the size → determination of the size corresponding to the optimum → determination of the parameters from the size.

SDMs based on the application of goal functions should be applied whenever significant changes in the properties of the dominant organisms are expected as a result of drastic changes in the forcing functions. But up to year 2000, this model type has been applied only about 13 times. We know that ecosystems and their organisms are adaptable, which implies that when predictions resulting from radical changes of forcing functions are required, it is recommended firstly to calibrate and validate the model using the observations from a sufficiently long period of time to uncover the dynamic of the state variables. The period may contain, for instance, some seasonal changes or parameters (sizes) that may allow us to test the structurally dynamic approach in parallel by the validation. If the structurally dynamic approach yields at least an equally good validation as does the fixed parameters approach, it seems feasible to apply the SDM approach for the development of prognoses. If the structurally dynamic approach cannot be tested, it is still recommendable to apply it for the development of prognoses, as we do know that ecosystems currently change their structure, but the prognoses should be used prudently.

IBM attempts to account for the enormous variability among individuals. Usually we apply one state variable to account for an average organism to represent a biological component. Thereby we violate the individuality of individuals. Darwinian selection is only possible if individuals have different properties. These differences are crucial in the survival of species. The average species may not be able to survive under the prevailing conditions, while some individuals with a better combination of properties such as a bigger size may be survivors. If the forcing functions are changed, it often becomes even clearer that the individuality of organisms is of utmost important for the reaction of the ecosystem. This is also the idea behind SDM — namely that ecosystems are adaptable to the steadily changed conditions. If an aquatic ecosystem is modeled by the use of a multispecies description but still with rigid parameters for each of the applied species, only one or two species will survive (see Nielsen[28]). A multispecies model with rigid parameters, i.e., without either a spectrum of properties as for IBM or with changeable parameters according to the prevailing conditions as for SDM, will not be able to describe properly the processes of ecosystems. A model based on average properties will in such a situation give completely wrong results, while IBM or SDM may be able to be in better accordance with the observations. They should therefore be applied as a modeling approach whenever it is of importance for the modeling results that the individuals have properties different from the average and that a current adaptation will always take place. This can be examined by varying the most sensitive properties (parameters) within realistic ranges and observing if the model results are decisive, for example, survival / not survival or abundant / scarce.

OOP should be mentioned in this context, although it may be considered a particular modeling technique and not another model type. OOP uses the concept of classes. One example of a class is the definition of a population, which is the basic building block for many ecological models. Populations are characterized by variables such as mean size, age, number, reproduction, growth, and mortality. Each type of population is unique, although there are many similarities, such as the above mentioned processes. We can therefore treat different classes of populations accordingly and need only to add those particular features that need to be different in the model context. The OOP defines different properties in different modules that can be used in the various classes. This short overview shows that it is a system based on model building blocks that makes a series of models more similar in structure and therefore easier to develop.

ANN is increasingly used to obtain a workable and good model on the basis of a huge but heterogeneous database from many different sites, when we want to know which factors (forcing functions) determine the composition (the state variables) of the examined type of ecosystem.

The model types presented above are practically applied model formulations, dependent on the problem, the data, the ecosystem, and the objectives of the modeler. They cover the great majority of the model types applied in modeling practice. Table 2 summarizes the characteristics of the various types mentioned above and gives guidance on the selection of model type. Often it may be more important to select the right type than to increase the complexity of the model. When, for instance, the structural dynamic changes actually take place, an increased complexity will not solve that problem. Similarly, if the variations of the individual properties are important for the description of the ecosystem reactions, only individual-based models can solve the problem satisfactorily.

Another recent discussion within ecosystem theory and ecological modeling is concerned with the possibilities of ecosystems showing chaotic and catastrophic behavior.

**TABLE 2**
**Overview of Presented Model Types**

| Model Type | Characteristics | Selection Criteria |
|---|---|---|
| Matrix representation | Gives linear relationships | Linear equations valid, age structure required |
| Static models | Give a good quantitative overview of steady state (average) situation | Applied in a data-poor situation where quantification is needed, but changes (e.g., seasonal) are not important |
| Fuzzy models | Give semi-quantitative results or just indication of ranges | Applied in a data-poor situation; semi-quantitative results sufficient |
| Representation by differential equations | Gives time and/or space variations | Good database needed |
| SDMs | Give variations of parameters as a function of time and/or space by expert knowledge or goal function | Prognoses under changed conditions needed; good database with some shifts in properties |
| IBMs | Consider the different properties of individuals | Applied where average properties (parameters) are insufficient |
| ANNs | Give a good correlation between forcing functions and state variables | Applied with a huge heterogeneous database; unfortunately not causal |
| Catastrophe theory | Explains a sudden change, but does not consider change in properties due to adaptation | Applied where a sudden change in the composition of the ecosystem is observed |

Chaos theory has raged throughout all sciences as a steppe fire during the last 2 decades. In has resulted in new insights, particularly into the behavior of systems. It is therefore obvious that an attempt should be made to use chaos theory in modeling, and the results of the considerations will be briefly discussed in the next section.

Sudden shifts are observed in ecosystems, although not very frequently. It has been demonstrated that these special cases of shifts can be described by catastrophe theory, a mathematical tool developed by Thom.

All eight model types presented in Table 2 have been applied in aquatic ecosystem modeling. The application is described in Table 3.

Last but not least, four focal recommendations on selection of a model are presented here as the clear rules (based on previous and recent experience) on which we base model development in the beginning of the 21st Century:

1. Remember that the model is only as reliable as its least reliable input. This entails that a balanced complexity of the submodels is recommended.
2. Keep the model as simple as possible and as complex as needed.
3. Remember that the most important outcome of the modeling effort may be a better understanding of the system, not necessarily a reliable, quantitative prediction. This implies that the modeler should attempt to develop a model with the right structure.
4. Maintain the system thinking. The model is not a correct representation of reality, but an attempt to describe important system features of the system–problem complex.

Models of ecosystems attempt to capture the characteristics of ecosystems. However, ecosystems differ from most other systems by being extremely adaptive, having the ability of self-organization, and having a large number of feedback mechanisms. The real challenge to modeling is: how can we construct models that are able to reflect these characteristics? Some

**TABLE 3**
**Model Applications**

| Model Type | Description of Extent of Use and Major Applications in Aquatic Ecosystem Modeling |
|---|---|
| Matrix models | Mainly used in population dynamic models |
| Static models | The software ECOPATH widely used to develop this type of model |
| Fuzzy models | Limited use but promising results where semiquantitative results are sufficient |
| Dynamic biogeochemical models | Widely used, but the shortcomings (do not consider adaptation and succession) have provoked new modeling types, for instance, SDMs |
| SDMs | Employed in a few case studies where expert knowledge is used and in 13 case studies where a goal function is used; promising results, but wider application needed before final conclusions can be made on the applicability |
| IBMs | Important approach in many cases, but still limited experience |
| ANNs | Increasing use to find good indicators for important state variables |
| Catastrophe theory | Limited use to cases where shifts in the composition of ecosystems are crucial |

recent developments have attempted to answer this question. If our models do not properly describe adaptation and possible shifts in species composition, the prognoses inevitably will be more or less incorrect.

## A PROMISING NEW CALIBRATION PROCEDURE

If they are based on a good database, aquatic ecosystem models often contain many parameters that make them very difficult to calibrate. In addition, as already discussed in connection with SDM, seasonal succession is observed in ecosystems. This is of particular importance in aquatic ecosystems because of their fast turnover rates. All modelers of aquatic ecosystems have, I am sure, felt the frustration of trying again and again to calibrate a relatively complex model without a good accordance between model results and observations. When we consider that the phytoplankton and zooplankton species are very different in the spring, summer, and fall, it is not surprising that we cannot get one set of parameters to fit to the model for the entire year. The problem is similar to the problem discussed above: how can we consider the important process of adaptation to the currently changed forcing functions in our models? It was therefore obvious to try to use for the phytoplankton and zooplankton parameters an optimization of the goal function exergy to perform the calibration instead of the difference between model results and observations. The size of the phytoplankton and zooplankton determining several parameters (three to four for each size) will of course facilitate the optimization. The other sensitive parameters, for instance, the release rate of nutrients from the sediment, can be calibrated in the normal way. The criteria could be best fit to the observations coupled with a return to the initial values year after year, if the forcing functions are the same year after year.

The described calibration approach has been tested with very promising results. The accordance between observations and model results in the calibration and in the validation phases was improved by a factor of two (see Jørgensen[29] and Jørgensen et al.[30]).

## SUMMARY AND PERSPECTIVES

The many new modeling approaches offer new possibilities for development of ecological models applicable as management tools and in system ecological research. Particularly the machine-learning techniques and SDMs seem to offer possibilities for a significant improvement of the model quality measured by the calibration and validation. ANN has been widely used to develop a model based upon a huge heterogeneous database but has the disadvantage that it does not lead to a causal model. By a combination with generic algorithms, it is possible, however, to build causality into this type of model.

Many of the new model types have had only a limited application, and it is therefore of importance to get a wider experience with these types of models to be able to evaluate their applicability. SDMs have until now shown promising results both by being able to predict structural changes and to obtain — in accordance with very recent results — a better calibration of aquatic ecosystem models due to the ability of this model type to account for succession. Further experience with this model type is therefore crucial.

Other possibilities that have not yet been examined are the development of hybrids. Can we unite ANN with IBM, or formulate generic algorithms that are based on the ecosystem properties applied in the development of ANNs? Also fuzzy techniques could be applied in combination with one of the other modeling types.

The biggest step forward in modeling will, however, come from a better understanding of ecosystems. An ecosystem theory is definitely being formed, as was agreed among 18 system ecologists at a meeting in Porto Venere and in Copenhagen in May–June of last year. It was also agreed that it was worthwhile to try to show that the theory could be applied to explain many ecological observations and rules (correlations). SDM is reflecting ecosystem properties, but many other properties may be built into our models in the future to limit the number of possible solutions and thereby to facilitate the formulation of processes, the calibration, and the validation.
